# Synthesis of Methoxy Amido Xanthate Ligand and Preliminary Rhenium-188 Radiolabeling Studies

**DOI:** 10.5812/ijpr-171671

**Published:** 2026-06-21

**Authors:** Ali Ashrfi Goudarzi, Mehdi Salehi Barough, Leila Moghaddam-Banaem, Saeed Kakaei, Fariba Johari Daha

**Affiliations:** 1 Department of Medical Radiation Engineering, Central Tehran Branch, Islamic Azad University, Tehran, Iran; 2 Nuclear Science and Technology Research Institute (NSTRI),School of Nuclear Fuel Cycle, Tehran, Iran; 3 Nuclear Science and Technology Research Institute (NSTRI), School of Radiation Application, Tehran, Iran

**Keywords:** ^188^Re, Radiolabeling, Methoxyamido Xanthate, Chelator

## Abstract

**Background:**

Rhenium-188 (^188^Re) is a high-energy β^-^-emitting radionuclide with favorable physical and chemical properties for targeted radionuclide therapy and theranostic applications. In this study, a novel sulfur- and nitrogen-containing chelator, methoxy amido xanthate (MAX), was synthesized and evaluated for efficient radiolabeling with ^188^Re. In a second step, radiolabeling of the alendronate bisphosphonate was assessed for potential theranostic applications in bone metastases.

**Objectives:**

This study aimed to synthesize and characterize the MAX ligand, optimize its radiolabeling with ^188^Re, and evaluate the association between the ^188^Re-MAX complex and alendronate.

**Methods:**

The ligand was prepared by reacting chloroacetamide with xanthate derivatives and was characterized using Fourier-transform infrared (FTIR) and nuclear magnetic resonance (NMR) spectroscopy. Radiolabeling was performed using generator-eluted sodium perrhenate (^188^ReO_4_^-^) under reducing conditions using stannous chloride and ascorbic acid. Key parameters affecting labeling efficiency, including ligand concentration, pH, reducing agent amount, and the effect of ascorbic acid as a stabilizing agent, were systematically optimized. Radiochemical purity was assessed by instant thin-layer chromatography (ITLC). In the second step, varying amounts of alendronate were added to ^188^Re-MAX under optimal conditions at 100°C for 30 minutes, and radiochemical purity was measured to determine the optimal alendronate amount.

**Results:**

Under optimal conditions (pH 5 - 6), radiochemical yields exceeding 98% were achieved at 100°C (80 - 100 mCi of ^188^Re, 12 mg MAX, 6 mg SnCl_2_, and 4 mg ascorbic acid) for ^188^Re-MAX. These findings indicate that the MAX ligand forms a stable complex with ^188^Re and represents a promising platform for the development of therapeutic and theranostic radiopharmaceuticals. For ^188^Re-MAX-alendronate, 96% radiochemical purity was achieved by adding 8 mg of alendronate to the mixture at pH 5 - 6.

**Conclusions:**

A novel MAX ligand was successfully synthesized and radiolabeled with ^188^Re under mild conditions. Alendronate was also labeled using ^188^Re-MAX. High radiochemical yields and purity were achieved by optimizing key labeling parameters. Given the favorable physical characteristics of ^188^Re and the strong chelation observed, the ^188^Re-MAX complex is a viable candidate for the development of therapeutic and theranostic radiopharmaceuticals. The ^188^Re-MAX complex demonstrated favorable radiochemical properties and warrants further biological evaluation, including toxicity, stability, biodistribution, and dosimetric studies, before therapeutic application can be considered.

## 1. Background

Radiopharmaceuticals have become indispensable tools in modern nuclear medicine, enabling both noninvasive diagnostic imaging and targeted radionuclide therapy. Among therapeutic strategies, bone-targeting agents have attracted particular attention because skeletal metastases are common in cancers such as prostate and breast carcinoma. In this context, bisphosphonates represent a well-established class of compounds with strong affinity for hydroxyapatite in bone tissue, enabling selective localization in areas of high bone turnover. Their chemical structure, characterized by a P-C-P backbone, provides excellent chelation capability for radiometals, making them suitable candidates for developing bone-seeking radiopharmaceuticals (1, 2).

Although technetium-99m (^99^mTc) remains the radionuclide of choice for single-photon emission computed tomography imaging, there is increasing interest in therapeutic radionuclides that can be paired with diagnostic isotopes in theranostic strategies (3-5).

Radiolabeled bisphosphonates have been extensively investigated for both imaging and therapy. Complexes labeled with ^99^mTc have long been used in bone scintigraphy, whereas therapeutic radionuclides such as rhenium-186 (^186^Re) and ^188^Re have shown promising results for palliating bone pain associated with metastatic disease. Among these radionuclides, ^188^Re is particularly attractive because of its high β^-^ energy (E_βmax_ ≈ 2.12 MeV), which enables effective tumor cell killing, and its simultaneous γ emission (155 keV), which allows imaging and dosimetric evaluation.

Despite these advantages, the development of stable and efficient rhenium-based radiopharmaceuticals remains challenging. Rhenium chemistry is more complex than technetium chemistry because of its multiple accessible oxidation states and relative resistance to reduction. Therefore, robust chelating systems capable of stabilizing rhenium in lower oxidation states are essential. Although bisphosphonates are effective for bone targeting, they may not always provide optimal coordination environments for rhenium, necessitating the incorporation of additional donor groups or hybrid ligand systems (6). In addition, ^188^Re is readily available from a long-lived tungsten-188/rhenium-188 (^188^W/^188^Re) generator system, offering logistical advantages similar to those of the widely used molybdenum-99/technetium-99m (^99^Mo/^99^mTc) generator (5).

The chemistry of rhenium closely resembles that of technetium; however, rhenium is generally more resistant to reduction and requires carefully optimized labeling conditions to achieve stable complex formation (7, 8). Therefore, the design of appropriate chelators is critical for successful radiolabeling and in vivo stability. Chelators containing soft donor atoms, such as sulfur and nitrogen, have demonstrated strong affinity for rhenium in low oxidation states (9).

In recent years, sulfur-containing ligands have emerged as promising candidates for rhenium coordination chemistry. Xanthate-based ligands are characterized by a strong metal-binding ability through sulfur donor atoms, which have high affinity for soft metal centers such as reduced rhenium species. These ligands have been widely studied in coordination chemistry and have demonstrated the ability to form stable complexes with transition metals. Their structural versatility also allows functional modification and the introduction of additional donor atoms, such as nitrogen and oxygen, to enhance chelation strength and kinetic stability.

Combining xanthate moieties with other functional groups offers a strategic approach to designing multifunctional ligands for radiopharmaceutical applications. By integrating nitrogen and oxygen donor atoms into a sulfur-rich framework, chelators can be created that form highly stable complexes with rhenium while maintaining favorable pharmacokinetic properties. Such hybrid systems may also be adapted for conjugation with biologically active molecules, including bisphosphonates, to achieve targeted delivery to specific tissues such as bone.

In this context, the development of novel ligands that combine the strong metal-binding properties of xanthates with additional coordinating functionalities represents a promising direction in radiopharmaceutical chemistry. The MAX ligand investigated in this study was designed to provide a multidentate coordination environment incorporating sulfur, nitrogen, and oxygen donor atoms. This structural arrangement is expected to enhance complex stability with ^188^Re and improve radiolabeling efficiency under mild conditions (10).

Bisphosphonates such as alendronate exhibit strong binding affinity for calcium-rich hydroxyapatite surfaces in bone and are widely used for targeting skeletal metastases. In radiopharmaceutical chemistry, bifunctional approaches that combine a radiometal chelator with a bisphosphonate group have attracted attention for theranostic applications. Alendronate was selected as a representative bisphosphonate model because of its strong affinity for hydroxyapatite crystals in bone tissue and its extensive use in skeletal-targeting applications. The phosphonate functional groups of alendronate provide high mineral-binding capability, making it suitable for investigation as a potential carrier for therapeutic radionuclides.

## 2. Objectives

In the present work, the synthesized MAX ligand was first radiolabeled with ^188^Re and subsequently associated with alendronate to investigate the feasibility of forming a bone-seeking therapeutic complex (11). By examining the coordination behavior of this novel ligand system, the study contributes to the ongoing development of effective and versatile radiopharmaceutical platforms for therapeutic and theranostic applications, with potential future integration into bone-targeting strategies involving bisphosphonate conjugates.

## 3. Methods

### 3.1. Materials

Chloroacetamide, carbon disulfide (CS_2_), potassium hydroxide, ethanol, methanol, stannous chloride dihydrate (SnCl_2_·2H_2_O), ascorbic acid, and all solvents were of analytical grade and were used without further purification. Alendronate sodium trihydrate, a clinically established nitrogen-containing bisphosphonate, was used as the bone-targeting moiety in this study. This compound was obtained in analytical-grade purity from a commercial pharmaceutical supplier and used without further purification. Sodium perrhenate (^188^ReO_4_^-^) was obtained by eluting a ^188^W/^188^Re generator with sterile normal saline from PARS-Isotope Company of Iran (12). The activity of ^188^ReO_4_^-^ was measured using a dose calibrator (Isomed, Germany) and was 500 - 600 mCi.

Ultraviolet (UV) spectra of the compounds were obtained using a Varian Cary 3 spectrometer. Radiochromatographic analysis was performed using silica gel ITLC chromatography paper from Agilent Technologies (United States). NMR analysis was performed using a Bruker DRX-300 MHz NMR spectrometer, and FTIR analysis was performed using a VECTOR 22 FTIR spectrometer.

The experiment was performed in 2 stages. In the first stage, the procedure was conducted with natural rhenium to assess the initial amounts of materials. In the second stage, the procedure was continued with ^188^Re. In the first stage, the amounts of chemical materials were selected as follows: ascorbic acid, 3 - 5 mg; SnCl_2_, 1 - 4 mg; alendronate, 5 - 25 mg; MAX, 2 - 7 mg; and pH, 4 - 6. The reaction temperature was selected between room temperature and 100°C.

### 3.2. Synthesis of Xanthate Intermediate

The xanthate compound was prepared as previously reported (13). Briefly, 100 g of KOH was added to a flask, and 300 mL of methanol at 65°C was added dropwise. After dissolution was complete, the reaction temperature was adjusted to 30°C, and 100 mL of CS_2_ was added. The formation of potassium xanthate was indicated by the appearance of a yellow precipitate, which was filtered, washed with cold methanol, recrystallized, and dried under vacuum (14). Potassium xanthate salt (1) was obtained as yellow crystals with a yield of 94%. The resulting material was crystallized from diethyl ether and hot ethanol and dried under a UV lamp for 1 day. Yellow crystals with a melting point of 224 - 226°C were formed.

### 3.3. Synthesis of Methoxy Amido Xanthate

The MAX ligand was synthesized by reacting the prepared xanthate intermediate with chloroacetamide in an aqueous medium. The reaction mixture was stirred under mild heating and monitored by thin-layer chromatography. Upon completion, the product was extracted with dichloromethane, dried over anhydrous sodium sulfate, and purified to obtain a white crystalline solid.

### 3.4. Ligand Characterization

Fourier-transform infrared spectroscopy was used to confirm the presence of characteristic functional groups, including amide (C=O) and xanthate (C=S) vibrations. Structural confirmation was further achieved using ^1^H and ^13^C NMR spectroscopy (14). At this step, UV spectroscopy was also performed to evaluate the labeling process.

### 3.5. Radiolabeling with ^188^Re

Radiolabeling was performed by adding generator-eluted sodium perrhenate (^188^ReO_4_^-^) to an aqueous solution of the MAX ligand. Stannous chloride was used as the reducing agent to convert rhenium to a lower oxidation state suitable for chelation. In selected experiments, ascorbic acid was added as a radiolytic stabilizer. The reaction mixture was incubated at 100°C for 20 - 30 minutes (8).

### 3.6. Association of ^188^Re-MAX With Alendronate

After radiolabeling the MAX ligand with ^188^Re, alendronate sodium was added as a bone-targeting bisphosphonate component. Different amounts of alendronate (5 - 25 mg) were evaluated to optimize complex formation and radiochemical stability. The reaction mixture was incubated at 100°C for 15 - 20 minutes under gentle stirring.

### 3.7. Optimization of Labeling Parameters

The effects of ligand concentration, pH (4-8), the amount of reducing agent, and the presence of ascorbic acid on labeling efficiency were systematically evaluated. Optimal conditions were defined as those yielding maximum radiochemical purity with minimal formation of free perrhenate or reduced hydrolyzed rhenium species (8).

### 3.8. Radiochemical Purity Assessment

Radiochemical purity was determined using ITLC-SG. Two mobile phases, acetone and saline, were used to separate free ^188^ReO_4_^-^, colloidal rhenium, and the ^188^Re-MAX complex. The radioactivity distribution was measured using a gamma counter, and radiochemical purity was expressed as a percentage of total activity.

## 4. Results

### 4.1. Ligand Characterization

**Figure 1.**
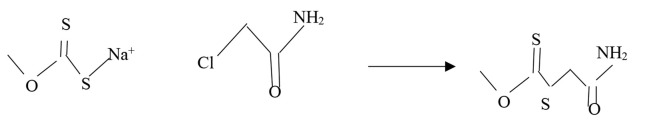
Schematic of the synthesis and structure of methoxy amido xanthate

The MAX ligand was prepared by reacting chloroacetamide with xanthate in water as the solvent. White crystalline precipitates with a melting point of 118 - 120°C were formed. The ligand contains oxygen, sulfur, and nitrogen atoms as lone-pair donor atoms. Its schematic representation is presented in Figure 1.

**Figure 2.**
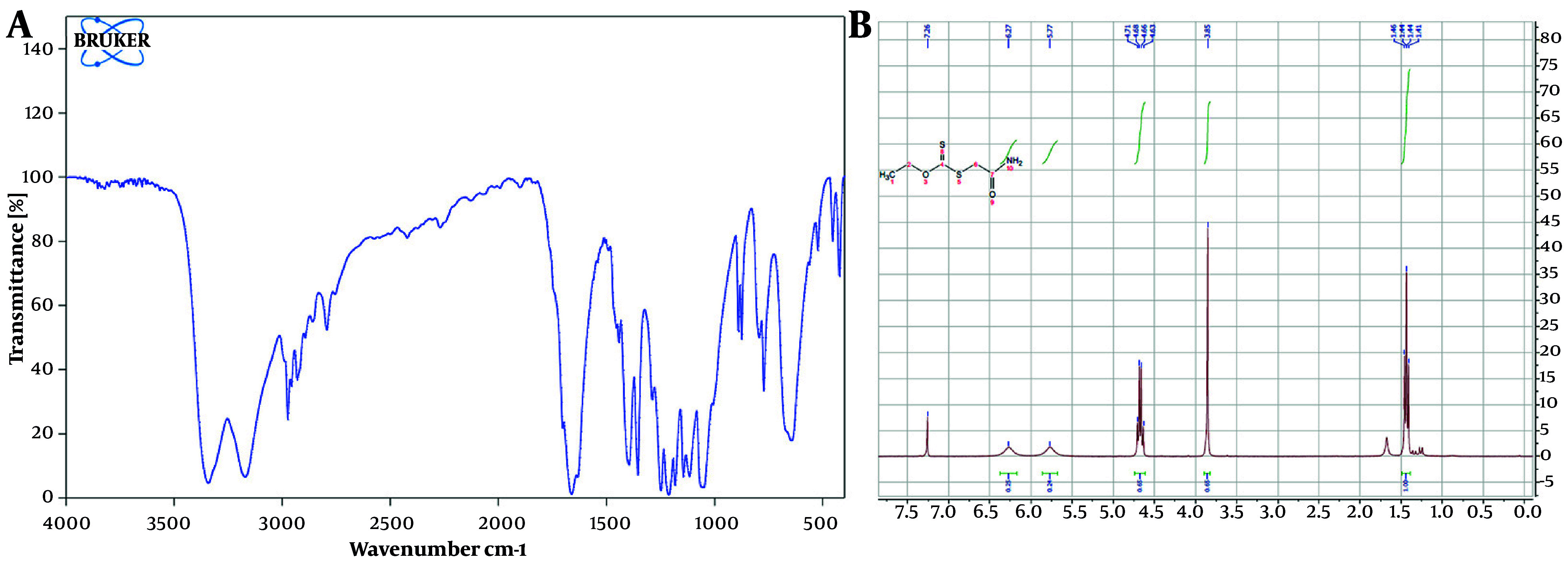
A, FTIR spectra of the synthesized MAX; B, NMR spectra of the synthesized MAX

The FTIR spectrum is shown in Figure 2A. The peak observed in the 2800 - 2900 cm^-1^ region was attributed to C-H stretching vibrations. The strong peak at 1650 cm^-1^ was attributed to the C=O stretching vibration of the amide group. Absorption peaks at 1100 cm^-1^ and 750 cm^-1^ corresponded to the asymmetric and symmetric stretching vibrations of CS_2_, respectively. The peak related to free amine functional groups (NH_2_) appeared at 3350 cm^-1^. In addition, the peak corresponding to C-N bond vibrations appeared at 1250 cm^-1^. These peaks indicate the successful synthesis of the MAX chelator.

The NMR spectrum is shown in Figure 2B. The peak at 1.44 ppm corresponded to the CH_3_ group connected to CH_2_. The peak at 3.85 ppm corresponded to CH_2_ bound to sulfur. Peaks at 5.77 and 6.27 ppm were related to the hydrogens of the NH_2_ group. In addition, the peak at 4.66 ppm was related to CH_2_ connected to CH_3_, and the peak at 7.26 ppm was related to the CDCl_3_ solvent. The NMR peaks confirmed the synthesis of the MAX chelating molecule (15).

### 4.2. Optimization of ^188^Re Labeling

Radiolabeling efficiency was strongly influenced by pH and reducing-agent concentration. Maximum labeling yields (> 90%) were achieved at pH 5 - 6, consistent with previously reported rhenium-based systems (6, 8). At lower pH values, protonation of donor atoms reduced chelation efficiency, whereas higher pH values promoted hydrolysis of reduced rhenium species.

Different alendronate amounts, ranging from 5 to 25 mg, were investigated to optimize the secondary association step with the ^188^Re-MAX complex. At low amounts (5 mg), incomplete association and lower radiochemical stability were observed. Increasing the alendronate amount improved radiochemical performance, with the best results obtained at approximately 8 mg, at which radiochemical purity exceeded 90%. Further increases above 10 mg did not significantly improve labeling efficiency and slightly reduced reproducibility in some experiments, possibly because of excess phosphonate competition or changes in solution composition. Therefore, 8 mg was selected as the optimal amount for subsequent experiments. The addition of ascorbic acid significantly enhanced radiochemical stability by reducing radiolysis, particularly at higher activity levels (16).

At this stage, alendronate labeling was also performed. After MAX synthesis and ^188^Re labeling, different amounts of alendronate, ranging from 5 mg to 20 mg, were added to ^188^Re-MAX (12 mg MAX + 80 - 100 mCi ^188^Re) to adjust the alendronate amount and achieve 96% radiochemical purity. The optimal amount was 8 mg of alendronate.

Reaction parameters, including ascorbic acid, SnCl_2_, pH, temperature, and alendronate, were evaluated by ITLC. The results are shown in Table 1.

**Table 1.** Effect of Ascorbic Acid, SnCl_2_, pH, Temperature, and Alendronate on Radiochemical Purity (80 - 100 mCi ^188^Re; N = 3)

MAX (mg)	Ascorbic acid (mg)	SnCl_2_ (mg)	pH	Alendronate (mg)	Temperature (°C)	Radiochemical purity	No.
**4**	3	3	4	5	Room temperature	10 ± 1%	3
**6**	4	4	4	10	Room temperature	10 ± 2%	3
**6**	3	4	4	5	100	50 ± 1.2%	3
**10**	5	4	5	20	100	40 ± 2%	3
**12**	4	6	5	10	100	80 ± 3%	3
**12**	4	6	5 - 6	8	100	95 - 96 ± 0.6%	3

The expected interaction mechanism is based on coordination and electrostatic association between the reduced ^188^Re-MAX core and the phosphonate-containing alendronate molecule. Because of the multidentate nature of bisphosphonates, the final product may consist of a ternary-associated complex and/or partially transchelated rhenium species.

### 4.3. UV Spectroscopy

**Figure 3.**
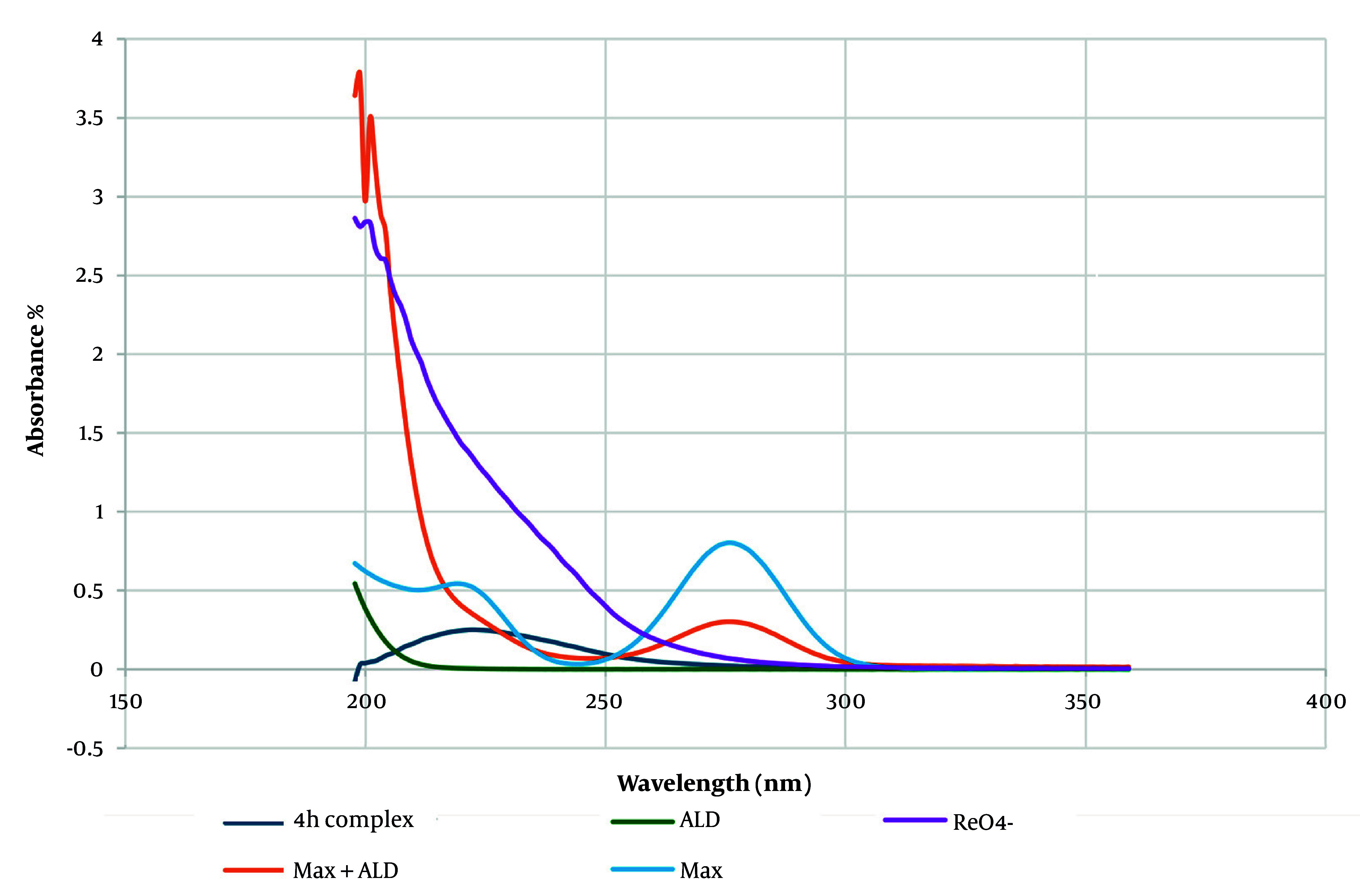
UV spectrum of ReO_4_^-^, alendronate, MAX, MAX + alendronate, and the complex (^188^Re + MAX + alendronate)

**Figure 4.**
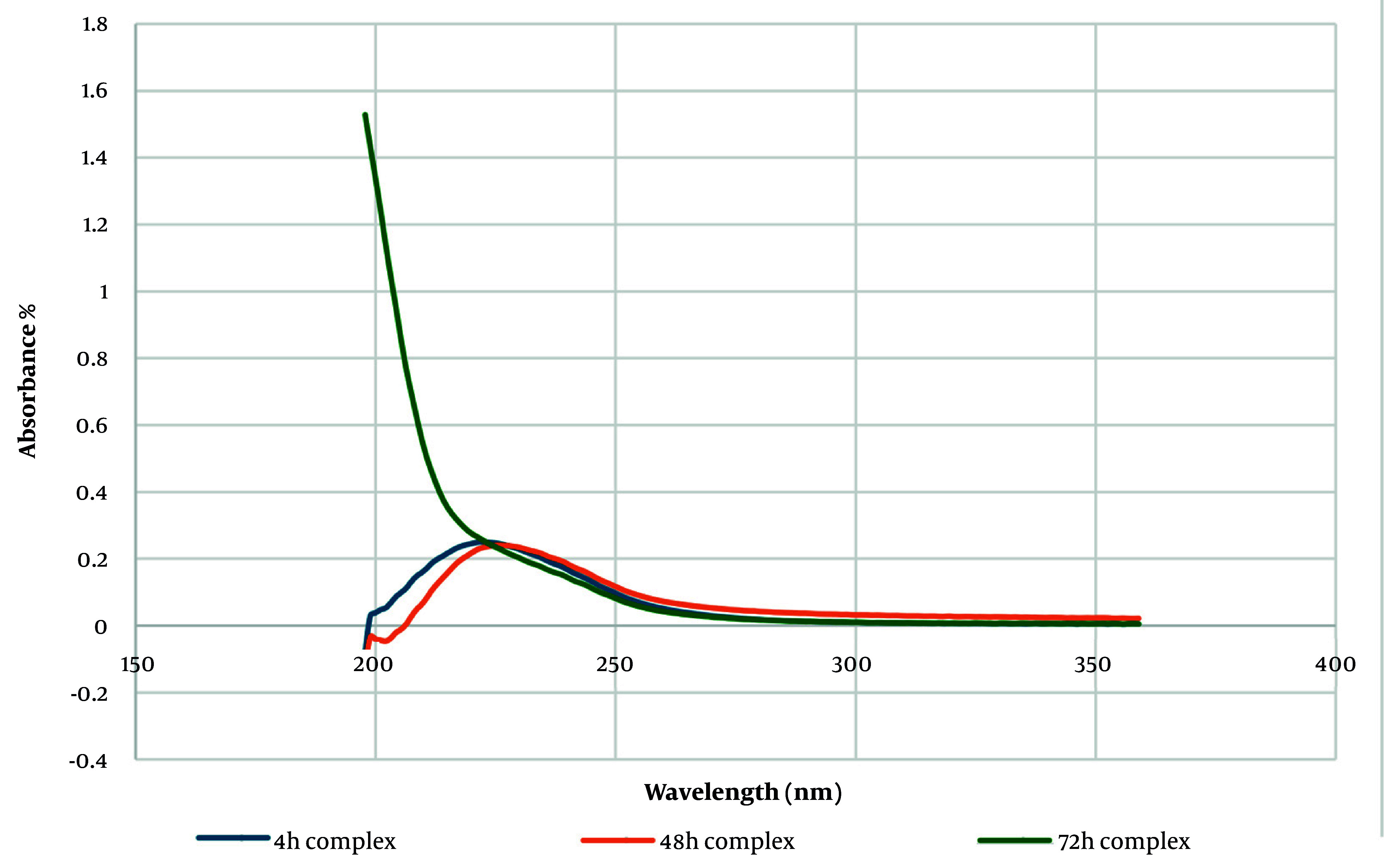
UV spectrum of the complex (^188^Re + MAX + alendronate) at 4, 48, and 72 hours

The UV-visible spectra are presented in Figures 3 and 4. Figure 3 shows the absorption spectra of natural ReO_4_^-^, MAX, alendronate, MAX + alendronate, and the complex at 4 hours.

In the MAX and MAX + alendronate spectra, a peak was observed at 275 nm. In the complex, this peak disappeared and shifted to 225 nm, as shown in Figure 4. This phenomenon was attributed to ligand-to-metal charge transfer from the ligand to the solvent in the complex and stabilized the bond between ReO_4_^-^ and MAX (17).

UV spectra were obtained at different time points, as shown in Figure 4. The complex remained stable for 48 hours after production, whereas degradation was evident at 72 hours. Stability up to 48 hours is comparable with the half-life of ^188^Re; therefore, ^188^Re-MAX was considered stable during its effective period.

### 4.4. Radiochemical Purity

**Figure 5.**
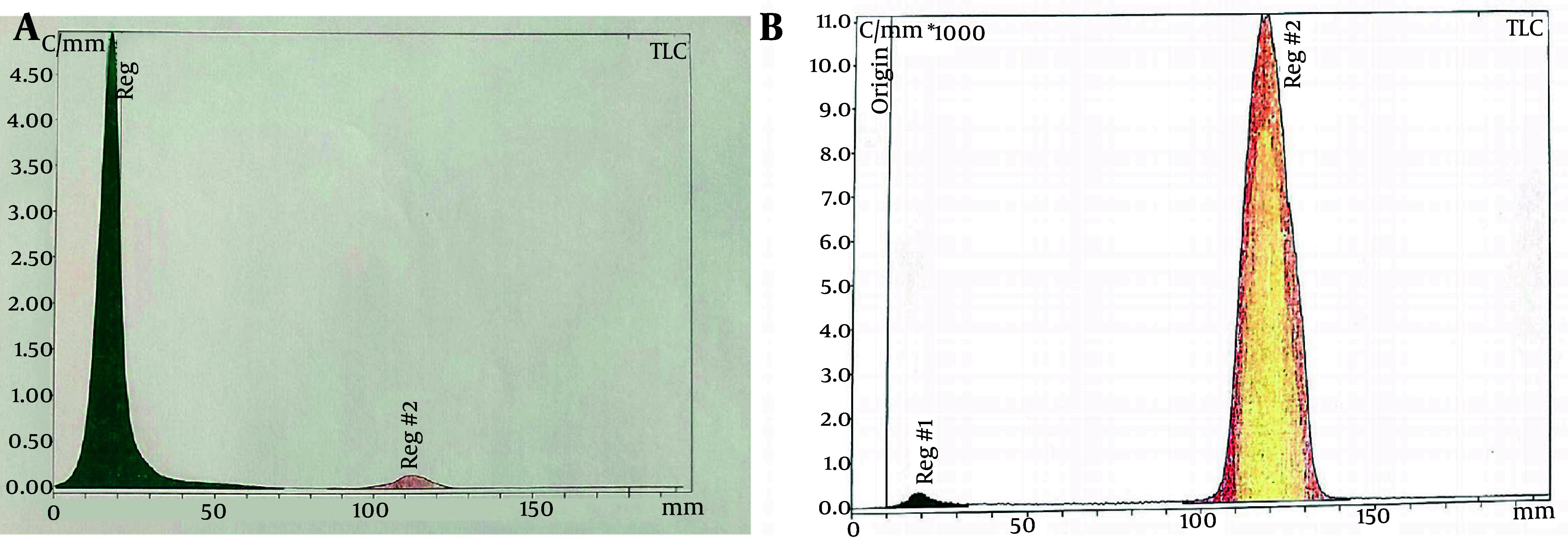
A, ITLC of the ^188^Re-MAX-alendronate complex with acetone as the mobile phase; B, ITLC of the ^188^Re-MAX-alendronate complex with 0.9% saline as the mobile phase

Figure 5A and B show the ITLC analysis of the complex using a two-solvent system consisting of acetone and saline. Figure 5A shows acetone as the mobile phase. The complex (R_f_ = 0) and ^188^Re_2_O_4_ (R_f_ = 0) remained at the spotting site, whereas free ^188^ReO_4_^-^ (R_f_ = 1) moved to the solvent front, allowing determination of the percentage of ^188^ReO_4_^-^. Figure 5B shows saline as the mobile phase. Free ^188^ReO_4_^-^ (R_f_ = 1) and the compound (R_f_ = 1) moved with the solvent front, whereas ^188^Re_2_O_4_ (R_f_ = 0) remained at the spotting site, allowing determination of the percentage of ^188^Re_2_O_4_. Radiochemical purity was calculated using Formula 1:

Formula 1:

%Radiochemicalpurity=100-%188ReO4--%188Re2O4

ITLC analysis demonstrated radiochemical purity exceeding 90% under the optimized conditions, with minimal free perrhenate detected. These results indicate the formation of stable ^188^Re-MAX and ^188^Re-MAX-alendronate complexes suitable for further biological evaluation and therapeutic application (1, 7). The R_f_ values for ^188^Re-MAX and ^188^Re-MAX-alendronate were the same.

Using Formula 1, the radiochemical purity was 98 ± 0.6% for ^188^Re-MAX and 96 ± 0.5% for ^188^Re-MAX-alendronate.

## 5. Discussion

The successful labeling of MAX with ^188^Re under mild conditions demonstrates the effectiveness of sulfur- and nitrogen-containing donor atoms in stabilizing reduced rhenium species. Compared with traditional rhenium chelation systems, which often require elevated temperatures or more complex coordination frameworks, the MAX ligand enabled efficient labeling with a high radiochemical yield. This finding is particularly significant given the relatively challenging reduction chemistry of rhenium compared with that of technetium. The optimal pH range of 5 - 6 reflects a balance between ligand deprotonation and the prevention of hydrolysis. The requirement for a controlled SnCl_2_ concentration further highlights the importance of precise reduction chemistry in rhenium labeling.

In this work, alendronate labeling remained stable for 48 hours, which is comparable with the half-life of ^188^Re. Therefore, ^188^Re-MAX-alendronate can be considered stable over its effective period, which is a promising feature for bisphosphonate labeling. Although biological stability studies were not included in this work, the high in vitro radiochemical purity and stability in the presence of ascorbic acid suggest that the complex may be suitable for further preclinical evaluation.

The present findings demonstrate that the MAX ligand can support efficient in vitro radiolabeling of ^188^Re under mild conditions and may provide a useful platform for future radiopharmaceutical development. However, the current evidence is limited to analytical and radiochemical observations, and additional biological stability and preclinical evaluation studies are required before therapeutic or theranostic applicability can be established. Although the toxicity of MAX was not assessed in this study, Schick et al. (18) reported that xanthate derivatives displayed antitumor activity against transformed fibroblasts and lymphoma cells in combination with monocarboxylic acids.

The incorporation of alendronate was intended to introduce potential bone-targeting capability through the high affinity of bisphosphonate groups for hydroxyapatite. However, direct evaluation of bone affinity was not performed in the present work. Hydroxyapatite-binding assays, serum stability studies, cellular uptake experiments, cytotoxicity assays, and in vivo biodistribution analyses are required to confirm skeletal targeting performance and biological stability. Therefore, the current study should be considered a preliminary radiochemical investigation, whereas biological validation remains the subject of future work.

### 5.1. Conclusions

Methoxy amido xanthate was successfully synthesized and radiolabeled with ^188^Re under mild conditions. Alendronate was also labeled with ^188^Re-MAX. Optimization of key labeling parameters resulted in high radiochemical yields and purity. Given the favorable physical properties of ^188^Re and the strong chelation observed, the ^188^Re-MAX complex demonstrated favorable radiochemical properties and warrants further biological evaluation, including toxicity, stability, biodistribution, and dosimetric studies, before therapeutic application can be considered. Future studies will focus on hydroxyapatite-binding evaluation, in vitro serum stability, biodistribution, and dosimetric assessment to confirm the bone-targeting and theranostic potential of the ^188^Re-MAX-alendronate system. Although noticeable degradation was observed at 72 hours, the complex remained stable for at least 48 hours, corresponding to approximately 2.8 physical half-lives of ^188^Re (T_1/2_ = 16.9 hours).

## Data Availability

The dataset presented in the study is available on request from the corresponding author during submission or after publication. The data are not publicly available.
